# Comparison of the pain-reducing effects of EMLA cream and of lidocaine tape during arteriovenous fistula puncture in patients undergoing hemodialysis: A multi-center, open-label, randomized crossover trial

**DOI:** 10.1371/journal.pone.0230372

**Published:** 2020-03-25

**Authors:** Keiji Fujimoto, Hiroki Adachi, Keita Yamazaki, Kanae Nomura, Atsushi Saito, Yuji Matsumoto, Kazunari Igarashi, Hiroko Uranishi, Suga Sakaguchi, Toshikazu Matsuura, Junko Imura, Kazuaki Okino, Kiyotaka Mukai, Yuki Okushi, Yu Kagaya, Yuko Tsuruyama, Keiichiro Okada, Nobuhiko Miyatake, Takatoshi Haraguchi, Yasuo Iida, Hitoshi Yokoyama

**Affiliations:** 1 Department of Nephrology, Kanazawa Medical University School of Medicine, Ishikawa, Japan; 2 Keiju Medical Center, Ishikawa, Japan; 3 Kanazawa Medical University Himi Municipal Hospital, Toyama, Japan; 4 Honma Hospital, Yamagata, Japan; 5 Anamizu General Hospital, Ishikawa, Japan; 6 Imura Clinic, Ishikawa, Japan; 7 Department of Mathematics, Kanazawa Medical University, Ishikawa Japan; Kaohsiung Medical University Hospital, TAIWAN

## Abstract

Arteriovenous fistula puncture pain is a serious problem for patients undergoing dialysis and a good indication for topical anesthetics. No previous study has compared lidocaine/prilocaine cream (EMLA) with lidocaine tape for pain relief during arteriovenous fistula puncture in patients undergoing maintenance hemodialysis. To this end, we conducted a multicenter randomized crossover study including 66 patients (mean age, 65.8 years; males, 57.6%) undergoing maintenance hemodialysis thrice/week. Subjects were assigned to Sequence EL (EMLA administration followed by lidocaine, with 1-week wash-out) or Sequence LE (reverse administration, first lidocaine then EMLA). All subjects completed the study. At each puncture site, 1 g EMLA (25 mg lidocaine + 25 mg prilocaine) or one sheet of lidocaine tape (18 mg lidocaine) was applied 1 h or 30 min prior to arteriovenous fistula puncture, respectively. The primary endpoint was puncture pain relief, which was measured using a 100-mm visual analog scale. The secondary endpoints included quality of life, which was measured by SF-36, and safety. EMLA produced a 10.1-mm greater visual analog scale improvement than lidocaine tape (P = 0.00001). However, there was no statistically significant difference in the quality of life between the two groups, and no significant carryover/period effect was observed in any analysis. Further, no drug-related adverse events were observed. Taken together, these results suggest that EMLA cream is superior to lidocaine tape for the relief of arteriovenous fistula puncture pain in patients undergoing maintenance hemodialysis.

**Trial registration:** University Hospital Medical Information Network Clinical Trials Registry (UMIN000027885).

## Introduction

Japan is the second country with the largest number of patients receiving chronic maintenance dialysis worldwide, after the United States [1]. The number has been increasing annually and exceeded 300,000 in 2012 [2]. For patients undergoing chronic maintenance hemodialysis, the pain associated with arteriovenous fistula (AVF) punctures during each dialysis session causes a great deal of stress [3–5]. In Japan, AVF accounts for a greater proportion of vascular access than in the United States (Japan, 96.8%; the United States, 55.5%) [6,7], and many patients undergo hemodialysis over a long period of time due to the small number of renal transplants; therefore, this issue represents an urgent problem. Furthermore, the number of patients who receive chronic maintenance hemodialysis is predicted to significantly increase worldwide [1]. Currently, the United States National Kidney Foundation has developed a “Fistula First” awareness campaign regarding vascular access [7], which strongly recommends AVF. Thus, AVF is predicted to become the main choice of vascular access globally, which makes AVF puncture pain an important issue for patients in Japan and worldwide.

In Japan, lidocaine tape is typically applied at the puncture site to alleviate pain during AVF puncture. However, in many cases, it is difficult to reach a sufficient anesthesia due to low skin permeability [8]. Lidocaine/prilocaine (EMLA) cream possesses superior skin permeability and therefore provides adequate anesthesia. Since its approval in Sweden in 1984, EMLA cream has been widely used across the world [9–19], and is currently approved in more than 80 countries. EMLA is indicated for relieving pain associated with injection or intravenous indwelling needle punctures and minor skin operations and is clinically indicated for pediatric patients. In Japan, EMLA was approved in January 2012 for uses related to “pain relief for skin laser therapy” and has been commercially available since May of the same year. Following approval for “relief of pain when puncturing an injection needle/venous indwelling needle” in June 2015, EMLA has been used for pain relief during AVF puncture in patients undergoing chronic maintenance hemodialysis. However, the pain relief effect offered by EMLA during AVF punctures in these patients has not been fully examined. It is unknown whether EMLA provides superior pain relief compared to lidocaine tapes, which are conventionally used in Japan. Therefore, in the present study, we compared the effects of pain alleviation achieved by EMLA and by lidocaine tapes during AVF puncture using superiority testing procedures.

## Methods

### Patients

The present study was conducted across six Japanese hemodialysis facilities (Kanazawa Medical University Hospital, Ishikawa; Anamizu General Hospital, Ishikawa; Keiju Medical Center, Ishikawa; Imura Clinic, Ishikawa; Kanazawa Medical University Himi Municipal Hospital, Toyama; and Honma Hospital, Yamagata) from June 2017 to September 2018. We recruited patients aged ≥20 years who were undergoing chronic maintenance hemodialysis three times per week via forearm AVF (autologous blood vessel) for vascular access and who were able to provide written informed consent for participation. After signing the informed consent, the patient was included in the study. We excluded 1) patients with allergies to local anesthetics, 2) patients with a history of contact dermatitis in response to topical medication, 3) patients with severe liver disease [aspartate aminotransferase (glutamic oxaloacetic transaminase) or alanine aminotransferase (glutamic pyruvic transaminase) levels ≥100 IU/L in the last periodic blood sample], 4) patients with methemoglobinemia, 5) patients with porphyria, 6) pregnant or lactating female patients, 7) patients with skin abnormalities in or around the puncture site, 8) patients with severe sensory disorders, 9) patients who participated in another clinical trial within 3 months prior to study drug administration, and 10) patients scheduled to move to another dialysis facility during the study period.

The present study was approved by the institutional review board of Kanazawa Medical University (No. M427), conducted in accordance with the ethical principles based on the Declaration of Helsinki, and registered in the University Hospital Medical Information Network Clinical Trials Registry (UMIN000027885).

### Randomization and blinding

In the present study, we conducted case registration using the central registration method and randomized assignment using a software cloud service (IRUKA System, Tokyo, Japan). The randomization method used age, sex, baseline visual analog scale (VAS) of Period 0 (P0), and the presence or absence of diabetes as adjustment factors for stochastic minimization. Stochastic minimization was consistent with the method described by Pocock and Simon [20]. The researchers at each facility recorded the eligible patients’ information, including adjustment factors for stochastic minimization, on the study registration form on the cloud system. Immediately after, the patients were assigned to two sequence groups by the cloud computer program, and the assignment results were automatically sent back to the researchers via e-mail. To ensure that the allocation was effectively concealed, the history of assignments of the entire research work was maintained by a researcher who was not involved in the patients’ enrollment, interventions, or data analysis. Blinding was difficult because the dosage forms of the two drugs were very different. Thus, the present study was conducted as an open-label study.

### Treatment compliance

The coinvestigators at each facility confirmed that the intervention was performed in accordance with the protocol for all patients.

### Study drugs

The investigational drug, EMLA (Sato Seiyaku, Tokyo, Japan), contained 25 mg of lidocaine and 25 mg of prilocaine in 1 g. The control drug, lidocaine tape (Yutoku Yakuhin, Saga, Japan), contained 18 mg of lidocaine in one sheet (180 mg of plaster).

### Interventions

AVF puncture requires the puncture of two sites in each hemodialysis session, one on the blood removal side and one on the blood return side. In the present study, the treatment intervention was performed using a topical anesthetic at two sites: one for blood removal prior to hemodialysis and one for the return of blood. According to the usage dose described in the package insert of each drug, EMLA (1 g) was applied using a dedicated fixation tape at each puncture site 1 h before the AVF puncture; lidocaine tape was applied at each site to be punctured 30 minutes before the AVF puncture. The diameter of the puncture needle was 16 gauge for all patients.

### Outcome measures

The primary endpoint of the present study was AVF puncture pain and was measured using a 100-mm straight line VAS, with the left end indicating minimal pain, “no pain at all,” and the right end indicating maximum pain, “worst pain I have experienced.” All patients indicated the intensity of their pain by drawing a slash on the line based on their perceived pain by intuition. The distance from the left end of the written slash was taken as the evaluation score. Two VAS scores, one for the blood removal side puncture and one for the return side puncture, were measured for each session of dialysis and the mean was used as the VAS score of one dialysis session. To prevent recall bias, patients marked the VAS scale immediately after the AVF punctures, and both patients and investigators were completely isolated at the time of VAS measurement.

The secondary endpoints included assessment of the quality of life (QOL) and safety evaluation. The QOL was evaluated using the following six subscales in the SF-36 Japanese version 2 scale, acute form [21–23] (symptoms over the last week): (1) role physical, (2) bodily pain, (3) vitality, (4) social functioning, (5) role emotional, and (6) mental health. These scores were converted into linear scores ranging from 0 (worst score) to 100 (best score). The safety of the drugs used was evaluated using side effect reports for each intervention.

### Study design

**Fig 1:**
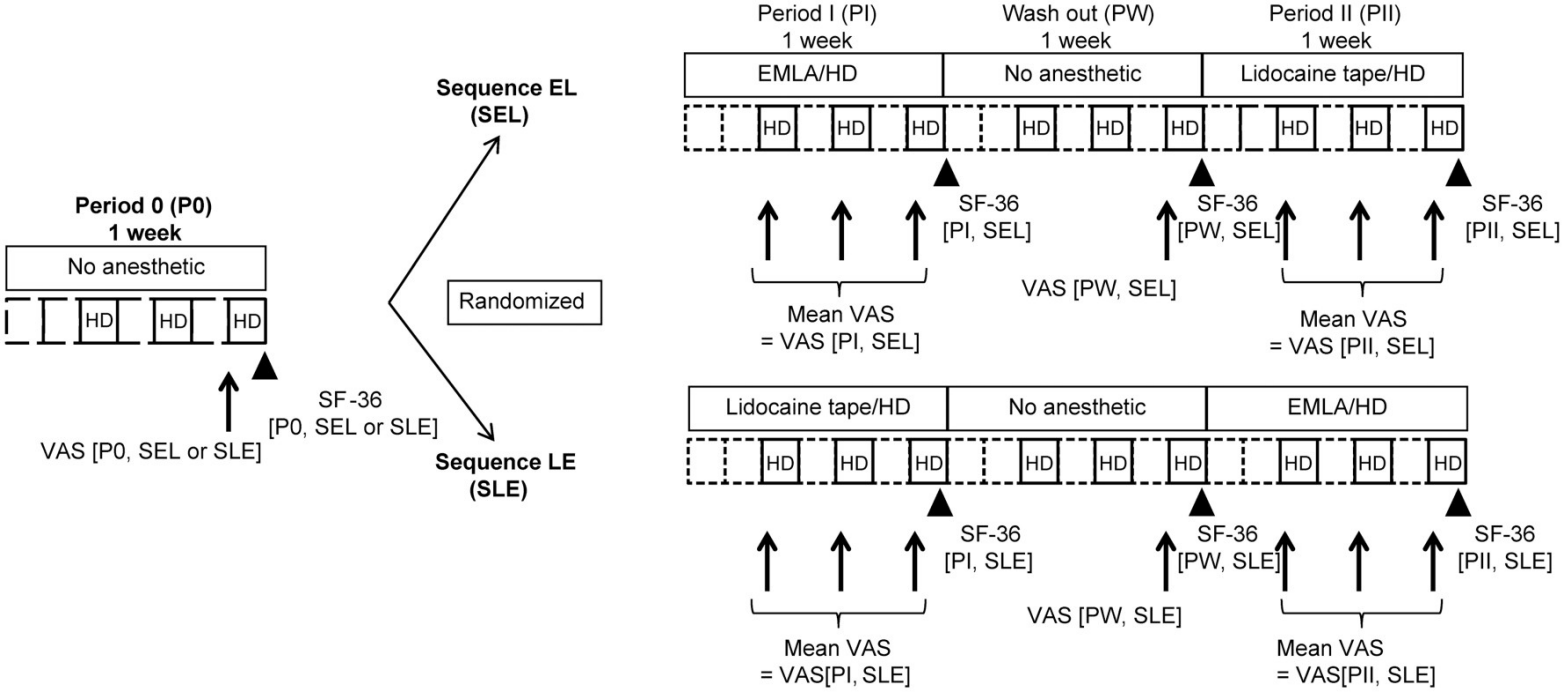
Study design overview. Abbreviations: HD, hemodialysis; VAS[Pi, Sj], VAS score of Period i in Sequence j (i = 0, I, W, II; j = EL, LE); SF-36[Pi, Sj], SF-36 score of Period i in Sequence j (i = 0, I, W, II; j = EL, LE); W, Wash-out. Arrows indicate VAS score measurements. The arrowhead indicates the SF-36 measurement. Squares with dotted lines indicate non-hemodialysis days and squares with solid lines indicate hemodialysis days.

The present study was a multicenter, open-label, randomized, active drug (lidocaine tape)-controlled, 2-group 2-phase crossover study. Patients were randomly assigned to Sequence EL (SEL) and treated with EMLA followed by lidocaine tape or to Sequence LE (SLE) and treated with lidocaine tape first followed by EMLA (Fig 1).

We measured the baseline VAS and SF-36 scores without a topical anesthetic at the end of the final hemodialysis of P0 and Wash-out period (PW); the measured value of P0 was defined as the baseline before the intervention of Period I (PI), and the measured value of PW was defined as the baseline before the intervention of Period II (PII).

PI and PII are intervention periods. We calculated the mean VAS score in three sessions of hemodialysis of PI and PII; the calculated value of PI was defined as VAS[PI, SEL or SLE], and the calculated value of PII was defined as VAS[PII, SEL or SLE].

Additionally, SF-36 score was measured at the end of the final hemodialysis of PI and PII; the measured value of PI was defined as SF-36 [PI, SEL or SLE], and the measured value of PII was defined as SF-36[PII, SEL or SLE].

Using the above measurement items, the amount of improvement in VAS and SF-36 before and after each treatment intervention (⊿VAS and ⊿SF-36) necessary for the analysis of the crossover study were calculated by each sequence (Table 1).

**Table 1:** The amount of VAS and SF-36 improvement before and after intervention by each sequence.

	The amount of VAS and SF-36 improvement by the PI intervention {⊿VAS[PI] and ⊿SF-36[PI]}	The amount of VAS and SF-36 improvement by the PII intervention {⊿VAS[PII] and ⊿SF-36[PII]}
SEL	⊿VAS[PI, SEL] = VAS[P0, SEL]–VAS[PI, SEL]	⊿VAS[PII, SEL] = VAS[PW, SEL]–VAS[PII, SEL]
	⊿SF-36[PI, SEL] = SF-36[PI, SEL]–SF-36[P0, SEL]	⊿SF-36[PII, SEL] = SF-36[PII, SEL]–SF-36[PW, SEL]
SLE	⊿VAS[PI, SLE] = VAS[P0, SLE]–VAS[PI, SLE]	⊿VAS[PII, SLE] = VAS[PW, SLE]–VAS[PII, SLE]
	⊿SF-36[PI, SLE] = SF-36[PI, SLE]–SF-36[P0, SLE]	⊿SF-36[PII, SLE] = SF-36[PII, SLE]–SF-36[PW, SLE]

### Selection of the wash-out period duration

Carryover effects were minimized by maintaining a wash-out period of approximately 1 week between the treatments in each Sequence (7 days until baseline VAS score of PW measurement, 10 days until initial drug use and initial measurement of VAS score in PII). The US FDA Bioequivalence Test Guidelines [24] recommend a wash-out period of at least five times the elimination half-life (t1/2) of the plasma drug concentration in the crossover method. In principle, healthy adults are to be selected as subjects in this test. However, the wash-out period of the pretreatment drug in the present study appeared to also be set in reference to this guidance.

In the Japanese Phase I trials [25, 26], the t1/2 of lidocaine was approximately 5.9 h, and the t1/2 of prilocaine was approximately 4.1 h, when 5 g of EMLA was applied at the back of the hands or forearms for 2 h. In addition, the t1/2 when two strips of lidocaine tapes were applied on the medial side of the upper extremities for 4 h was approximately 1.7 h. The t1/2 values of both drugs were measured using higher doses and/or longer lengths of application time than in the present study. Based on these data, we presumed that approximately 30 h is required for wash-out of these drugs. Furthermore, since the subjects of the present study were patients undergoing hemodialysis rather than healthy individuals, the wash-out period was set to be sufficiently long, taking into consideration the reduced renal drug clearance in these patients.

### Statistics

All statistical analyses were performed using Stat Flex Ver6 (Artech Co., Ltd., Osaka, Japan).

#### Sample size

No previous study has provided the information on the variances of VAS score amelioration from baseline due to the treatments. Therefore, the sample size was calculated by the intermediate analysis of the first 30 samples enrolled after the start of the present study. Thus, the variance in VAS score improvement among subjects treated with EMLA and among those treated with lidocaine tape was estimated to be 400 mm^2^ in each case. The covariance between subjects for both treatments, the within-subject variance of VAS score improvement by EMLA, and the within-subject variance of VAS score improvement by lidocaine tape were estimated to be 100 mm^2^ each. An approximate 10-mm difference has been proposed as the minimum clinically significant difference (MCSD) in VAS score for acute pain in previous studies [27–30]. Therefore, approximately 64 patients (32 for each Sequence group) were planned to be recruited for this study, to assure at least 80% statistical power to detect a 10-mm difference in the average VAS score improvement at a 5% significance level.

#### Comparison of patient characteristics between sequence groups

Comparisons for age, VAS score, and SF-36 score between sequences were performed using an unpaired Student’s t-test due to the normal distribution of the data. The comparison for hemodialysis vintage between sequences was performed by the Mann–Whitney U-test due to the non-normal data distribution. The normality of continuous variables was assessed by Shapiro-Wilk’s test. The comparison of distributions among sequences was tested using chi-squared test or Fisher’s exact test.

#### Primary endpoint analysis by crossover method and SF-36 score analysis method

A standardized crossover analysis method was used for the primary endpoint [31,32]. The carryover effect was examined by comparing the mean value of SEL time sum (PI + PII) with the mean value of SLE time sum (PI + PII), i.e., examination was performed by comparing {⊿VAS[PI, SEL] + ⊿VAS[PII, SEL]} with {⊿VAS[PI, SLE] + ⊿VAS[PII, SLE]} using unpaired t-test. The period effect was examined by comparing the mean value/2 of SEL time difference (PI–PII) and the mean value/2 of SLE time difference (PII–PI), i.e., examination was performed by comparing {⊿VAS[PI, SEL]–⊿VAS[PII, SEL] } / 2 and {⊿VAS[PII, SLE]–⊿VAS [PI, SLE]} / 2 using an unpaired t-test. The treatment effect was examined by comparing the mean value/2 of SEL time difference (PI–PII) and the mean value/2 of SLE time difference (PI–PII), i.e., examination was performed by comparing {⊿VAS[PI, SEL]–⊿VAS[PII, SEL]} / 2 and {⊿VAS[PI, SLE]–⊿VAS[PII, SLE] } / 2 using unpaired t-test. The significance probability of a carryover effect and period effect was P < 0.10 (two-tailed test), and the significance probability of a treatment effect was P < 0.05 (two-tailed test).

Regarding the secondary endpoints, a comparison of the change in SF-36 subscores between the two treatments was performed in the same way as for the primary endpoint, i.e., the carryover, period, and treatment effects were examined by comparing {⊿SF-36[PI, SEL] + ⊿SF-36[PII, SEL]} with {⊿SF-36[PI, SLE] + ⊿SF-36[PII, SLE]}, {⊿SF-36[PI, SEL]–⊿SF-36[PII, SEL]} / 2 with {⊿SF-36[PII, SLE]–⊿SF-36 [PI, SLE]} / 2, and {⊿SF-36[PI, SEL]–⊿SF-36[PII, SEL]} / 2 with {⊿SF-36[PI, SLE]–⊿SF-36 [PII, SLE]} / 2 between Sequence groups using unpaired t-tests.

## Results

### Study population

**Fig 2:**
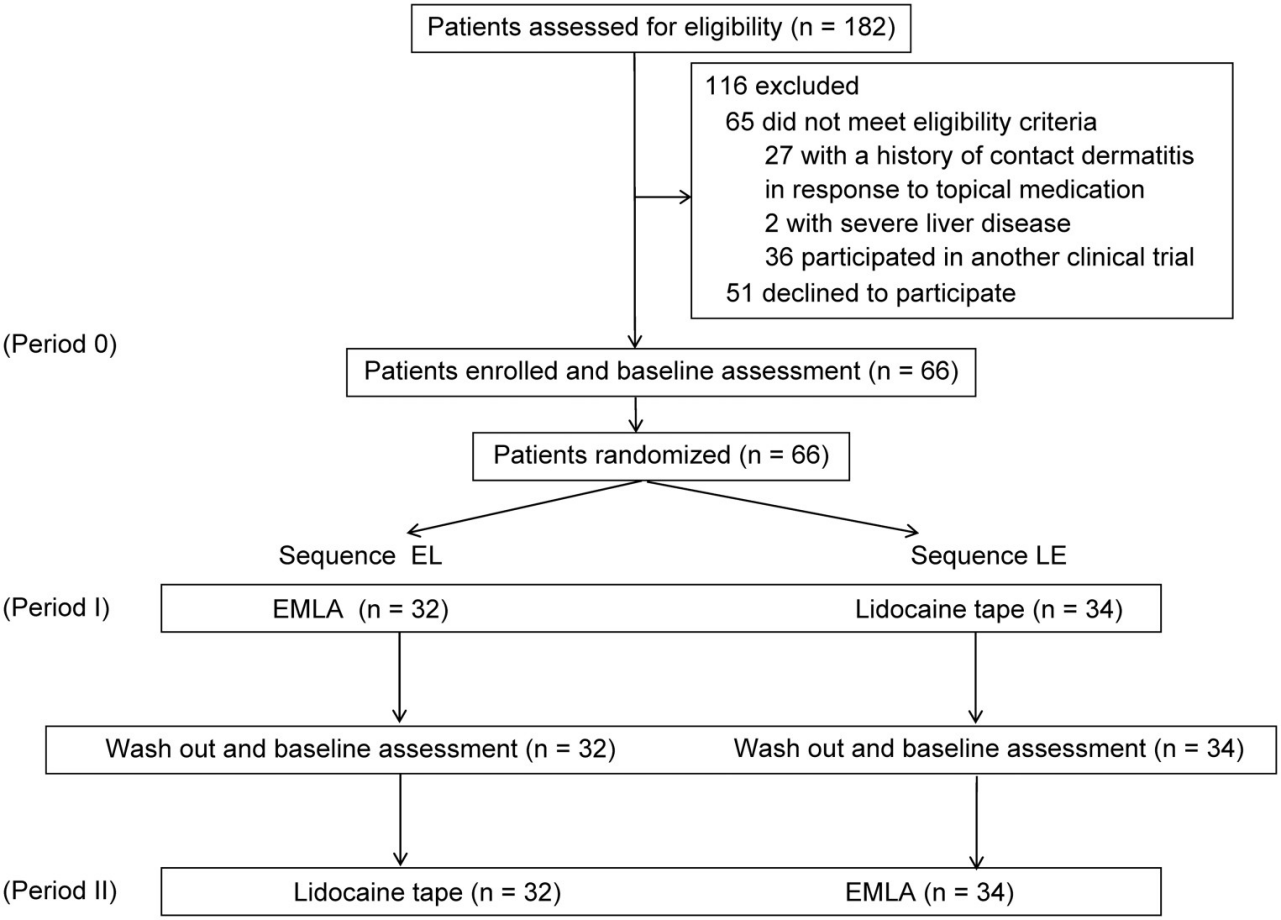
Study flow diagram. All study subjects completed the study and were included in the statistical analysis of outcomes.

The recruitment of participants ended when we were able to secure the number of patients needed for analysis (at least 32 patients per Sequence group). In total, 182 patients were screened for eligibility, 66 of whom met the eligibility criteria and were randomized between June 2017 and September 2018 (Fig 2). All randomized patients completed the trial. In accordance with the randomization procedure, 32 patients were assigned to SEL, and 34 patients were assigned to SLE. SEL patients were treated with EMLA cream followed by lidocaine tape, and SLE patients were treated with lidocaine tape followed by EMLA cream. Our analysis followed the principle of intention to treat analysis.

The baseline characteristics of the patients in the present study are shown in Table 2. There were no significant differences between groups regarding age, sex, dialysis vintage, prevalence of diabetes mellitus, degree of AVF puncture pain (VAS score at P0, that is the baseline VAS score before the intervention of PI; VAS score at PW, that is the baseline VAS score before the intervention of PII) without administration of topical anesthetic, or scores of both baseline SF-36 subscales (role physical, bodily pain, vitality, social functioning, role emotional, and mental health). On the other hand, there was difference in the distribution of dialysis facilities between sequences. The baseline VAS score of both Sequence groups was relatively low (P0 average, 29.8 mm; PW average, 29.2 mm) and the baseline VAS values for one fifth of patients (21.2% of P0, 22.7% of PW) were below 10 mm.

**Table 2:** Baseline characteristics of patients.

Characteristics	SEL EMLA followed by Lidocaine tape (n = 32)	SLE Lidocaine tape followed by EMLA (n = 34)	*P* value
Age (mean years ± SD)	66.2 ± 10.7	65.5 ± 10.6	*0*.*78*
Sex (male %)	20 (62.5%)	18 (52.9%)	*0*.*43*
Hemodialysis vintage [months, median (IQR)]	51.5 (10.0–140.5)	78.0 (11.0–147.0)	*0*.*68*
Diabetes	20 (37.5%)	23 (32.4%)	*0*.*66*
Dialysis facilities			
A	2 (6.2%)	0 (0%)	*0*.*049*
B	6 (18.8%)	9 (26.5%)	
C	1 (3.1%)	5 (14.7%)	
D	17 (53.1%)	9 (26.5%)	
E	3 (9.4%)	9 (26.5%)	
F	3 (9.4%)	2 (5.8%)	
VAS score at P0 (mean ± SD)	26.8 ± 19.7	32.7 ± 20.8	*0*.*24*
VAS score at PW (mean ± SD)	29.8 ± 25.3	28.5 ± 20.8	*0*.*81*
SF-36 score at P0 (mean ± SD)			
Role physical	74.0 ± 24.8	76.3 ± 29.1	*0*.*74*
Bodily pain	65.6 ± 23.0	75.9 ± 23.0	*0*.*07*
Vitality	61.3 ± 24.0	54.6 ± 27.1	*0*.*29*
Social functioning	82.8 ± 22.8	76.1 ± 30.7	*0*.*32*
Role emotional	74.5 ± 28.6	80.6 ± 30.6	*0*.*40*
Mental health	73.9 ± 16.3	69.0 ± 21.1	*0*.*30*
SF-36 score at PW (mean ± SD)			
Role physical	75.4 ± 25.2	76.1 ± 23.9	*0*.*90*
Bodily pain	69.1 ± 21.2	75.7 ± 24.2	*0*.*24*
Vitality	64.6 ± 24.2	57.9 ± 22.3	*0*.*24*
Social functioning	84.0 ± 20.9	79.4 ± 23.2	*0*.*40*
Role emotional	79.9 ± 22.5	75.0 ± 27.8	*0*.*43*
Mental health	75.3 ± 18.1	69.4 ± 19.9	*0*.*21*

Abbreviations: VAS, 100-mm visual analog scale

Comparisons for age, VAS score, and SF-36 score between sequences were performed using unpaired t-test. The comparison for hemodialysis vintage between sequences was performed using Mann–Whitney *U*-test. The comparison for the distribution of dialysis facilities between sequences was performed using Fisher’s exact test. Comparisons for other categorical variables between sequences were performed using Chi-square test.

### Reduction in pain

The results for the reduction in pain are shown in Table 3. EMLA produced a VAS score improvement that was 10.1 mm [95% confidence interval (CI): 5.9–14.2 mm] greater than that produced by lidocaine tape (P = 0.00001). There was no significant carryover effect or period effect noted (P = 0.64 and P = 0.46, respectively). No significant center effect on VAS improvement by each drug was observed (S1 Fig). Furthermore, even after adjusting for the allocation factor used in the minimization method (sex, age, baseline VAS, presence / absence of diabetes) by multiple regression analysis, the difference in intervention drugs (EMLA or lidocaine tape) was a significant predictor of VAS improvement in PI (S1 Table) and PII (S2 Table).

**Table 3:** Comparison of improvement in visual analog scale (VAS) score between EMLA and lidocaine tape.

	⊿VAS[PI] (mm)	⊿VAS[PII] (mm)	Carry over effect	Period effect	Treatment effect
SEL: EMLA followed by Lidocaine tape (n = 32)	⊿VAS[PI, SEL] = 18.9 ± 18.2	⊿VAS[PII, SEL] = 7.3 ± 16.4	*P* = 0.64	*P* = 0.46	*P* = 0.00001
SLE: Lidocaine tape followed by EMLA (n = 34)	⊿VAS[P I, SLE] = 10.4 ± 11.7	⊿VAS[PII, SLE] = 19.0 ± 17.4			EMLA treatment effect—Lidocaine tape treatment effect = 10.1 mm (95% CI: 5.9–14.2 mm)

Abbreviations: CI, confidence interval; EMLA: lidocaine/prilocaine cream.

The data are presented as mean ± standard deviation (SD). The significance probability of the carryover effect and period effect was *P* < 0.10, and the significance probability of the treatment effect was *P* < 0.05.

### Quality of life

The results for the QOL assessment are shown in Table 4. No statistically significant difference was observed in the treatment effect between EMLA and lidocaine tape regarding changes from baseline SF-36 subscale scores (Role physical, P = 0.97; Bodily Pain, P = 0.99; Vitality, P = 0.96; Social functioning, P = 0.37; Role emotional, P = 0.11; and Mental health, P = 0.95). No significant carryover effect or period effect was observed in these analyses.

**Table 4:** Comparison of changes in SF-36 scores from baseline between sequences.

Subscale of SF-36	Sequence	⊿SF-36 [PI]	⊿SF-36 [P II]	Carryover effect *P*	Period effect *P*	Treatment effect *P*
Role physical	EL	1.2 ± 22.8	2.7 ± 24.7	0.66	0.68	0.97
	LE	−0.4 ± 17.9	0.9 ± 17.9			
Bodily pain	EL	2.7 ± 23.6	1.3 ± 23.1	0.79	0.76	0.99
	LE	1.6 ± 27.8	0.4 ± 18.9			
Vitality	EL	2.0 ± 15.8	−0.2 ± 11.8	0.62	0.39	0.96
	LE	3.3 ± 17.7	0.9 ± 12.8			
Social functioning	EL	3.5 ± 20.1	4.7 ± 9.4	0.51	0.59	0.37
	LE	8.7 ± 25.5	3.0 ± 12.5			
Role emotional	EL	6.0 ± 26.5	0 ± 14.5	0.89	0.99	0.11
	LE	−0.5 ± 15.2	5.4 ± 26.9			
Mental health	EL	1.1 ± 13.4	−1.4 ± 12.8	0.18	0.32	0.95
	LE	4.7 ± 18.7	2.5 ± 12.9			

The data are presented as mean ± standard deviation (SD). The significance probability of the carryover effect and period effect was set at *P* < 0.10, and the significance probability of the treatment effect at *P* < 0.05.

### Safety

No adverse events due to either drug were observed.

## Discussion

The results of the present study demonstrate that EMLA significantly reduces AVF puncture pain compared to lidocaine tape. However, there is no definite consensus concerning MCSD of VAS pain score; thus, it is difficult to definitively conclude that the observed 10.1-mm (95% CI: 5.9–14.2 mm) difference in VAS score improvement between the two treatments in the present study is clinically significant.

The MCSDs proposed by four major studies on MCSD using VAS pain scores are 9 mm (95% CI: 6–13 mm) [28], 13 mm (95% CI: 10.0–16 mm) [27], 13 mm (95% CI: 10–17 mm) [30], and 12 mm (95% CI: 9–15 mm) [29]. Stratified analysis according to the severity of baseline pain showed that MCSD values were 11 mm (95% CI: 4–18 mm) for patients with mild pain at baseline (VAS ≤ 30 mm), 14 mm (95% CI: 10–18 mm) for those with moderate pain (31 mm ≤ VAS ≤ 69 mm), and 10 mm (95% CI: 6–14 mm) for those with severe pain at baseline (VAS ≥ 70 mm) [29]. These proposed MCSD values are similar to the difference in VAS score improvement between EMLA and lidocaine tape, observed in the present study. Furthermore, one fifth of patients in this study had no room for VAS score improvement of ≥ 10 mm because their baseline VAS values were < 10 mm. Thus, there might be a clinically significant difference in the treatment effect of these two drugs.

As most nerve endings in the skin are present in the dermis, topical anesthetics need to pass through the stratum corneum and reach the dermis to achieve their intended anesthetic effect. The stratum corneum has a layered structure of keratinocytes; intercellular lipids are present between keratinocytes and play a role as a barrier against water-soluble substances [33]. Therefore, in order for a local anesthetic to reach the dermis via the stratum corneum, a high concentration of a hydrophobic local anesthetic has to be contained in oil droplets of the formulation. Lidocaine and prilocaine exist as solids at room temperature, but once they are mixed equimolarly, their melting point is lowered, and the mixture becomes a liquid at room temperature [34]. Taking advantage of this property, the quantity of solubilizing agent (oil) added is reduced, and a high concentration of anesthetic is incorporated into the EMLA oil droplets [35]. Based on an adaption of these formulations, it appears that EMLA possesses superior skin permeability and sufficient anesthetic effect; data supporting the superior anesthetic effect in clinical practice have been reported mainly in the fields of dermatology, pediatrics, and anesthesiology [9–19, 36–38]. However, only few studies have provided high-level evidence on the effect of EMLA on pain inhibition during AVF puncture in patients under chronic maintenance hemodialysis. Only one randomized controlled study (RCT) showed that EMLA is more effective than placebo and cooling spray [39]. Furthermore, to the best of our knowledge, there are no RCTs comparing the pain relief effects of conventional lidocaine tape and EMLA for AVF puncture. Thus, the present study is the first RCT to demonstrate that EMLA is superior to lidocaine tape in reducing AVF puncture pain. In addition, safety was confirmed without any serious adverse event due to the administration of either drug.

The present study has several advantages. First, it shows external validity as it included patients from six hemodialysis facilities of different sizes. Second, outcome analysis was performed by randomization that could be maintained since no patients dropped out of the study. Third, we adopted a crossover design that avoided problems associated with individual differences, which have been considered to obscure differences in treatment effects in parallel group studies due to VAS and SF-36 scores being subjective indicators [32]. Fourth, the sample size design in this study was considered to be generally appropriate because the sample size calculated by the final analysis of all samples was about 60, which was close to the initially forecast number. Specifically, it was estimated based on the following values. The variance in VAS score improvement among subjects treated with EMLA was 310 mm^2^, and the variance in VAS score improvement among those treated with lidocaine tape was estimated to be 202 mm^2^ in each case. The covariance between subjects for both treatments was 112 mm^2^, and the within-subject variance of VAS score improvement by EMLA and the within-subject variance of VAS score improvement by lidocaine tape were both estimated to be 225 mm^2^. The difference in treatment effects of both drugs was 10.1mm in VAS.

However, the present study has several limitations. First, there was no placebo control, and thus, it was not possible to compare the treatment group with a non-treatment group. Second, the internal validity of the study is limited because it was not blinded. Third, the study’s generalizability is limited due to the small sample size. Fourth, the acute form of the SF-36 scale reflects the symptoms of 1 week, and we did not observe a significant difference in short-term QOL change between the two treatments. However, QOL changes due to long-term use of these drugs is unclear. Thus, it is necessary to examine whether the QOL amelioration effect differs between the two drugs after long-term drug use or when the standard version of SF-36 scale is used (review period of 1 month). Fifth, as the included patients were patients with renal disease, we cannot rule out the possibility that the SF-36 scale, a comprehensive QOL scale, did not sufficiently allow for the extraction of QOL changes after the therapeutic intervention. Generally, comprehensive QOL scales measure disease symptom characteristics; however, they are less informative and often less sensitive to changes in health conditions over time than disease-specific QOL scales. Therefore, additional investigations are necessary using renal disease-specific QOL scales, such as the KDQOL-SF scale [40].

## Conclusion

The results of the present RCT point to the superiority of EMLA cream to lidocaine tape in terms of AVF puncture pain alleviation in patients undergoing chronic maintenance dialysis.

## Supporting information

S1 Fig Comparison of VAS improvement by both treatment among the dialysis facilities. There were no significant differences in the amount of improvement in VAS by the administration of both drugs among the 6 dialysis facilities [(a) EMLA(n = 66), p = 0.06; (b) Lidocaine tape (n = 66), p = 0.31; Kruskal-Wallis test].
(TIF)

S1 Table The factors that influenced the VAS improvement in PI from baseline. (DOC)

S2 Table The factors that influenced the VAS improvement in PII from baseline. (DOC)

S1 File CONSORT 2010 checklist. (DOC)

S2 File CONSORT 2010 flow-diagram. (DOC)

S3 File Research protocol. (DOCX)
