# Supplementary figures and images for: Comparison of the pain-reducing effects of EMLA cream and of lidocaine tape during arteriovenous fistula puncture in patients undergoing hemodialysis: A multi-center, open-label, randomized crossover trial
Source: PLoS One. 2020 Mar 25;15(3):e0230372. doi: 10.1371/journal.pone.0230372 (PMC7094835; doi:10.1371/journal.pone.0230372)

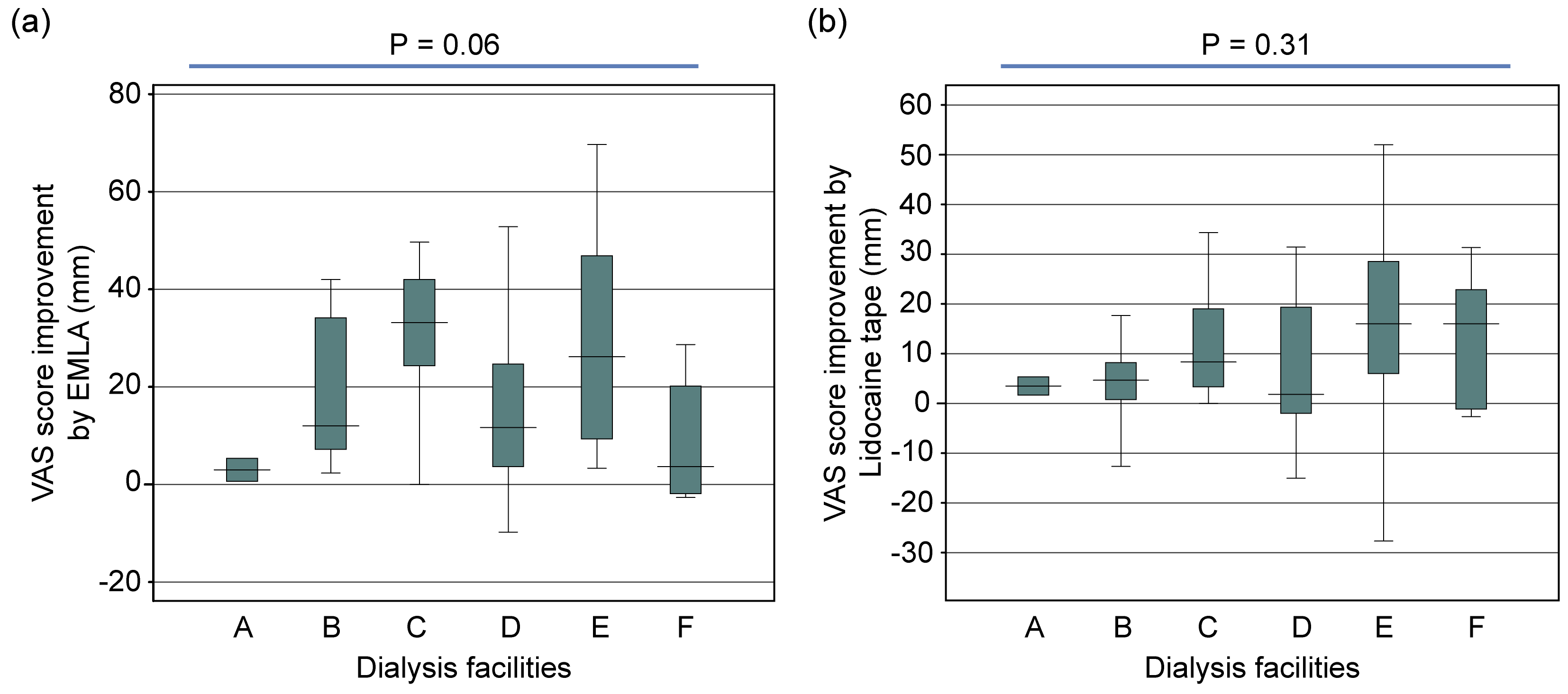

Supplement: S1 Fig — There were no significant differences in the amount of improvement in VAS by the administration of both drugs among the 6 dialysis facilities [(a) EMLA(n = 66), p = 0.06; (b) Lidocaine tape (n = 66), p = 0.31; Kruskal-Wallis test]. (TIF) [file pone.0230372.s001.tif]
