# Supplementary material for: Comparison of the pain-reducing effects of EMLA cream and of lidocaine tape during arteriovenous fistula puncture in patients undergoing hemodialysis: A multi-center, open-label, randomized crossover trial
Source: PLoS One. 2020 Mar 25;15(3):e0230372. doi: 10.1371/journal.pone.0230372 (PMC7094835; doi:10.1371/journal.pone.0230372)
Supplement: S1 Table — (DOC) [file pone.0230372.s002.doc]

**S1 Table. Factors that influenced the VAS improvement in PI from baseline.**

Object variable: ⊿VAS[PI]

| Explanatory variables | β | Stdβ | *P* value |
| --- | --- | --- | --- |
| Age | －0.07 | －0.05 | *0.51* |
| Sex (male) | 1.28 | 0.04 | *0.59* |
| VAS[P0] | 0.60 | 0.79 | *＜0.00001* |
| Diabetes | －2.39 | －0.07 | *0.34* |
| Treatment (EMLA) | 12.12 | 0.39 | *＜0.00001* |

Abbreviations: PI, Period I; ⊿VAS[PI], the improvement value of VAS

score by PI treatment; VAS[P0], VAS score in P0; β, partial regression coefficient; stdβ, standard partial regression coefficient

After adjusting for sex, age, VAS[P0], presence/absence of diabetes by multiple regression analysis, the difference in intervention drugs (EMLA or lidocaine tape) was a significant predictor of ⊿VAS[PI].
