# Supplementary material for: Comparison of the pain-reducing effects of EMLA cream and of lidocaine tape during arteriovenous fistula puncture in patients undergoing hemodialysis: A multi-center, open-label, randomized crossover trial
Source: PLoS One. 2020 Mar 25;15(3):e0230372. doi: 10.1371/journal.pone.0230372 (PMC7094835; doi:10.1371/journal.pone.0230372)
Supplement: S2 Table — (DOC) [file pone.0230372.s003.doc]

**S2 Table. Factors that influenced the VAS improvement in PII from baseline.**

Object variable: ⊿VAS[PII]

| Explanatory variables | β | Stdβ | *P* value |
| --- | --- | --- | --- |
| Age | －0.05 | －0.03 | *0.75* |
| Sex (male) | 5.58 | 0.16 | *0.13* |
| VAS[P0] | 0.45 | 0.52 | *＜0.00001* |
| Diabetes | －6.16 | －0.17 | *0.11* |
| Treatment (EMLA) | 9.16 | 0.26 | *0.01* |

Abbreviations: PII, Period II; ⊿VAS[PII], the improvement value of VAS

score by PII treatment; VAS[P0], VAS score in P0; β, partial regression coefficient; stdβ, standard partial regression coefficient

After adjusting for sex, age, VAS[P0], presence/absence of diabetes by multiple regression analysis, the difference in intervention drugs (EMLA or lidocaine tape) was a significant predictor of ⊿VAS[PII].
