# Supplementary material for: Comparison of the pain-reducing effects of EMLA cream and of lidocaine tape during arteriovenous fistula puncture in patients undergoing hemodialysis: A multi-center, open-label, randomized crossover trial
Source: PLoS One. 2020 Mar 25;15(3):e0230372. doi: 10.1371/journal.pone.0230372 (PMC7094835; doi:10.1371/journal.pone.0230372)
Supplement: S2 File — (DOC) [file pone.0230372.s005.doc]

**CONSORT 2010 Flow Diagram**

**Allocation**

**Analysis**

**Follow-Up**

**Enrollment**

**Sequence A**

**Sequence B**

Assessed for eligibility (n= 66)

Excluded (n= 0)

♦  Not meeting inclusion criteria (n=0)

♦  Declined to participate (n=0)

♦  Other reasons (n=0)

Analysed (n=32)
♦ Excluded from analysis (give reasons) (n=0)

Lost to follow-up (give reasons) (n=0)

Discontinued intervention (give reasons) (n=0)

Allocated to intervention (n= 32)

♦ Received allocated intervention (n=32)

♦ Did not receive allocated intervention (give reasons) (n=0)

Lost to follow-up (give reasons) (n=0)

Discontinued intervention (give reasons) (n=0)

Allocated to intervention (n=34)

♦ Received allocated intervention (n=34)

♦ Did not receive allocated intervention (give reasons) (n=0)

Analysed (n=34)
♦ Excluded from analysis (give reasons) (n=0)

Randomized (n=66)
