# Supplementary material for: Comparison of the pain-reducing effects of EMLA cream and of lidocaine tape during arteriovenous fistula puncture in patients undergoing hemodialysis: A multi-center, open-label, randomized crossover trial
Source: PLoS One. 2020 Mar 25;15(3):e0230372. doi: 10.1371/journal.pone.0230372 (PMC7094835; doi:10.1371/journal.pone.0230372)
Supplement: S3 File — (DOCX) [file pone.0230372.s006.docx]

Comparative study of EMLA cream and lidocaine tape on the reduction of the pain at arteriovenous fistula puncture in hemodialysis patients: A multicentre study.

**Study Protocol**

Short title: Pain relief during arteriovenous fistula puncture

Study Chair: Keiji FUJIMOTO, Department of Nephrology, Kanazawa Medical University

Study Coordinator: Keiji FUJIMOTO, Department of Nephrology, Kanazawa Medical University

Address: 1-1 Daigaku, Uchinada, Kahoku, Ichikawa 920-0293

Tel: 076-286-2211 (extension 8448)

Fax: 076-286-2786

E-mail: k-2210@kanazawa-med.ac.jp

Department of Nephrology, Kanazawa Medical University

Version 3.0

Created April 17, 2017

**Contents**

[1. Purpose **エラー! ブックマークが定義されていません。**](#_Toc480461239)

[2. Background and Scientific Rationale 5](#_Toc480461240)

[2.1. Background **エラー! ブックマークが定義されていません。**](#_Toc480461241)

[2.2. Scientific Rationale **エラー! ブックマークが定義されていません。**](#_Toc480461242)

[3. Drug Information **エラー! ブックマークが定義されていません。**](#_Toc480461243)

[4. Subjects **エラー! ブックマークが定義されていません。**](#_Toc480461244)

[5. Eligibility Criteria **エラー! ブックマークが定義されていません。**](#_Toc480461245)

[5.1. Selection Criteria **エラー! ブックマークが定義されていません。**](#_Toc480461246)

[6. Registration and Allocation **エラー! ブックマークが定義されていません。**](#_Toc480461247)

[6.1. Patient Registration Procedure **エラー! ブックマークが定義されていません。**](#_Toc480461249)

[6.2. Allocation Method and Allocation Adjustment Factors **エラー! ブックマークが定義されていません。**](#_Toc480461250)

[7. Study Design **エラー! ブックマークが定義されていません。**](#_Toc480461251)

[7.1. Content of Crossover Study **エラー! ブックマークが定義されていません。**](#_Toc480461252)

[7.2. Criteria for Dose and Schedule Changes **エラー! ブックマークが定義されていません。**](#_Toc480461253)

[7.3. Discontinuation of Treatment 11](#_Toc480461254)

[7.4. Concomitant Treatment and Supportive Therapy 11](#_Toc480461255)

[7.5. Post-Treatment **エラー! ブックマークが定義されていません。**](#_Toc480461256)

[8. Parameters to be Monitored, Tested, and Reported, and Schedule 12](#_Toc480461257)

[8.1 Parameters to be Monitored and Tested and Treatment Information to be Reported 12](#_Toc480461258)

[8.2. Schedule for Monitoring, Testing, and Reporting 13](#_Toc480461259)

[9.1. Target Enrollment **エラー! ブックマークが定義されていません。**](#_Toc480461260)

[9.2. Study Period **エラー! ブックマークが定義されていません。**](#_Toc480461261)

[10. Evaluation and Reporting of Adverse Events **エラー! ブックマークが定義されていません。**](#_Toc480461262)

[10.1. Definition of Adverse Events **エラー! ブックマークが定義されていません。**](#_Toc480461263)

[10.2. Evaluation and Reporting of Adverse Events **エラー! ブックマークが定義されていません。**](#_Toc480461264)

[10.3. Expected Adverse Events 14](#_Toc480461265)

[10.4. Urgent Reporting and Treatment of Adverse Events 15](#_Toc480461266)

[11. Cost to Study Subjects and Anticipated Risks and Benefits 16](#_Toc480461267)

[11.1. Cost to Study Subjects and Anticipated Risks **エラー! ブックマークが定義されていません。**](#_Toc480461268)

[11.2. Anticipated Benefits to Study Subjects 16](#_Toc480461269)

[11.3. Overall Assessment and Measures to Minimize Cost and Risks 16](#_Toc480461270)

[12. Matters to be Reported to the Directors of Study Sites and Reporting Method 16](#_Toc480461271)

[12.1. Reporting by Investigators and Subinvestigators 16](#_Toc480461272)

[12.2. Reporting by the Principal Investigator 16](#_Toc480461273)

[13. Definitions of Endpoints **エラー! ブックマークが定義されていません。**](#_Toc480461274)

[13.1. Primary Endpoint **エラー! ブックマークが定義されていません。**](#_Toc480461275)

[13.2. Secondary Endpoint 17](#_Toc480461276)

[14. Statistical Discussion **エラー! ブックマークが定義されていません。**](#_Toc480461277)

[15. Monitoring **エラー! ブックマークが定義されていません。**](#_Toc480461278)

[15.1. Conduct of Monitoring 19](#_Toc480461279)

[15.2. Actual Monitoring Procedure 19](#_Toc480461280)

[15.3. Matters to be Monitored 20](#_Toc480461281)

[16. Ethical Matters **エラー! ブックマークが定義されていません。**](#_Toc480461282)

[17. Handling of Personal Information エラー! ブックマークが定義されていません。](#_Toc480461283)

18. Study Costs

[18.1. Source of Funding and Financial Relationships 22](#_Toc480461284)

[18.2. Costs Associated with the Study 22](#_Toc480461285)

[18.3. Compensation for Health Damage 22](#_Toc480461286)

[19. Deviations from the Protocol **エラー! ブックマークが定義されていません。**](#_Toc480461287)

[19.1. Deviations from or Changes to the Study Protocol 22](#_Toc480461288)

[19.2. Revisions to the Study Protocol 23](#_Toc480461289)

[20. Termination or Early Completion of the Study 23](#_Toc480461290)

[20.1. Completion of the Study 23](#_Toc480461291)

[20.2. Early Termination of the Study 23](#_Toc480461292)

[21. Handling of Materials Associated with Study Materials 24](#_Toc480461293)

[22. Ownership of Study Results and Publication of Effects 24](#_Toc480461294)

[23. Study Organization **エラー! ブックマークが定義されていません。**](#_Toc480461295)

[23.1. Study Sites **エラー! ブックマークが定義されていません。**](#_Toc480461296)

[23.2. Principal Investigator **エラー! ブックマークが定義されていません。**](#_Toc480461297)

[23.3. Study Coordinator **エラー! ブックマークが定義されていません。**](#_Toc480461298)

[23.4. Statistical Analysis Manager 25](#_Toc480461299)

[23.5. Data Center and Data Manager 25](#_Toc480461300)

[23.6. Monitoring **エラー! ブックマークが定義されていません。**](#_Toc480461301)

[24. Support Desk **エラー! ブックマークが定義されていません。**](#_Toc480461302)

[25. References **エラー! ブックマークが定義されていません。**](#_Toc480461303)

1. **Purpose**

The purpose of this study is to carry out a positive-control, two-treatment, two-period crossover trial of the analgesic effect of lidocaine/propitocaine cream (brand name: EMLA Cream) compared with that of lidocaine tape (brand name: Youpatch Tape) during arteriovenous fistula puncture in chronic maintenance hemodialysis patients.

1. **Background and Scientific Rationale**

## Background

Pain during arteriovenous fistula puncture is a major reason for diminished quality of life (QOL) in chronic maintenance hemodialysis patients, and in Japan, where few patients receive kidney transplants, the unavoidable necessity of long-term maintenance dialysis after the start of hemodialysis makes it a compelling issue.

Efforts have been made to relieve pain during arteriovenous fistula puncture by applying lidocaine tape to the puncture site, but poor skin penetration means that in many cases its anesthetic effect is insufficient.^1)^ Because lidocaine/propitocaine cream penetrates the skin well, providing a good anesthetic effect, since its approval in Sweden in 1984 it has come to be widely used worldwide, and has currently been approved in more than 80 countries, where it is used for a variety of indications such as pain relief during injections and intravenous catheter placement and minor skin operations, as well as for children.^2) – 14)^ In Japan, it was approved in January 2012 for use as pain relief during skin laser irradiation therapy, and was launched on the market in May that year. In June 2015, it was also approved for use as pain relief during injections and intravenous catheter placement, since when EMLA Cream has been used to relieve pain during arteriovenous fistula puncture in chronic maintenance hemodialysis patients. However, the analgesic effect of lidocaine/propitocaine cream during arteriovenous fistula puncture in chronic maintenance hemodialysis patients has yet to be fully investigated, including overseas, and it is unclear whether or not its analgesic effect is superior to that of the lidocaine tape that has conventionally been used. This study (a multicenter, joint, nonblinded, nonrandomized, positive-controlled, two-treatment, two-period crossover trial) is a trial of the superiority of the analgesic effect during arteriovenous fistula puncture in chronic maintenance hemodialysis patients of lidocaine/propitocaine cream compared with lidocaine tape.

## Scientific Rationale for the Study

As most cutaneous nerve endings are located in the dermis, topical local anesthetics must pass through the stratum corneum to the dermis to exert an anesthetic effect. The stratum corneum has a structure consisting of layers of corneocytes, with intercellular lipids between these corneocytes acting as a barrier to water-soluble substances. To ensure that local anesthetics can pass through the stratum corneum and reach the dermis, formulations must therefore contain high concentrations of hydrophobic local anesthetics in the emulsified fraction.

Lidocaine/propitocaine cream utilizes the property that when lidocaine and propitocaine, both of which are solids at room temperature, are mixed in equimolar amounts their melting points decrease and they become liquid at room temperature.^15)^ This means that less solvent (oil) need be added, enabling the production of a formulation containing a high concentration of anesthetic in the emulsified fraction.^16)^ These modifications to the formulation should give lidocaine/propitocaine cream excellent skin penetration providing an adequate anesthetic effect, and data confirming its outstanding effectiveness as an anesthetic in clinical practice have been reported, mainly in the fields of dermatology, pediatrics, and anesthesiology.^2)–14)^ However, few studies providing high-level evidence have been conducted of its analgesic effect during arteriovenous fistula puncture in chronic maintenance hemodialysis patients, with only one overseas randomized comparative trial reporting that lidocaine/propitocaine cream has a more effective analgesic effect than placebo or cold spray.^17)^ Although lidocaine/propitocaine cream is thus expected to provide effective pain relief during arteriovenous fistula puncture in chronic maintenance hemodialysis patients, more evidence must be obtained. No randomized trial has previously been conducted to compare the analgesic effect of conventional lidocaine tape and lidocaine/propitocaine cream during arteriovenous fistula puncture. The present study is a superiority trial designed as a positive-control randomized comparative trial to evaluate the analgesic effect of lidocaine/propitocaine cream compared with lidocaine tape as a comparative control, with the objective of demonstrating whether lidocaine/propitocaine cream has a superior analgesic effect during arteriovenous fistula puncture than does lidocaine tape, or whether the reverse is true.

1. **Drug Information**

Overview of Investigational Drugs

Two investigational drugs will be used: the test drug [lidocaine/propitocaine cream (brand name: EMLA Cream)] and the control drug [lidocaine tape (brand name: Youpatch Tape)]. The test drug is approved for pain relief during injection needle/ pain relief during injections and intravenous catheter placement, and the control drug is approved for pain relief during intravenous catheter placement. Their use for pain relief during arteriovenous fistula puncture is covered by health insurance.

Test drug: Lidocaine/propitocaine cream (brand name: EMLA Cream)

Control drug: Lidocaine tape (brand name: Youpatch Tape)

Composition of test drug [lidocaine/propitocaine cream (brand name: EMLA Cream)]

| Content of the active principles (in 1 g) | JP Lidocaine 25 mg  Propitocaine 25 mg |
| --- | --- |
| Excipients | Polyoxyethylene hydrogenated castor oil, carbomer, pH regulator |

Composition and properties of control drug [lidocaine tape (brand name: Youpatch Tape)]

| Content of the active principle | 1 sheet (paste 180 mg) contains JP lidocaine 18 mg |
| --- | --- |
| Excipients | Styrene-isoprene-styrene-block copolymer, cycloaliphatic saturated hydrocarbon resin, liquid paraffin, one other component |
| Properties | Transdermal patch consisting of a colorless, semitransparent paste on a light brown to brown, flexible fabric support and liner |
| Size | 30.5 mm × 50.0 mm |

Dosage and Administration of Investigational Drugs

Test drug [lidocaine/propitocaine cream (brand name: EMLA Cream)]: 1 g of the agent is applied to a single location at the intended puncture site using special fixation tape 1 h before arteriovenous fistula puncture. After the agent has been left in place for 1 h, the tape is removed and the agent is wiped off. Vascular puncture is performed after thorough cleaning and disinfection.

Control drug [lidocaine tape (brand name: Youpatch Tape)]: 1 sheet of the agent is applied to a single location at the intended puncture site 30 min before arteriovenous fistula puncture. The sheet is removed after it has been left in place for 30 min. Vascular puncture is performed after thorough disinfection.

1. **Subjects**

Chronic maintenance hemodialysis patients aged ≥20 years who request topical drug use for pain relief during arteriovenous fistula puncture.

The pain score during arteriovenous fistula puncture is evaluated using a visual analog scale (VAS).

1. **Eligibility Criteria**

## Selection Criteria

1. Chronic maintenance hemodialysis patients undergoing hemodialysis 3 times a week
2. Age ≥20 years
3. Using an arteriovenous fistula (autologous vessels) for vascular access for hemodialysis
4. Informed consent provided voluntarily by the subject himself or herself in writing after a full explanation of study participation

Patients who meet conditions 1)–4) above who request the use of a topical drug for pain relief during arteriovenous fistula puncture

- 1. Exclusion Criteria

1. Allergy to local anesthetic
2. History of contact dermatitis in reaction to a topical drug
3. Serious liver disease [AST (GOT) or ALT (GPT) ≥100 IU/L in most recent regular blood test]
4. Methemoglobinemia
5. Porphyria
6. Pregnancy or lactation
7. Skin disorder at puncture site
8. Serious sensory disorder
9. Participation in another clinical study (clinical trial) within 3 months prior to the start of investigational drug administration
10. Considered by the Principal Investigator or another investigator to be ineligible as a study subject

[Rationale] 1)–6) For reasons of safety, 7)–10) for reasons of the effect on efficacy analysis or safety

1. **Registration and Allocation**

## Patient Registration Procedure

1. The Principal Investigator or another investigator obtains written consent from patients who meet the eligibility criteria.
2. Patient registration (using the central registration method) and randomization are conducted using a cloud–based allocation service system (Iruka System Co., Ltd., Tokyo). The results of this allocation are automatically sent to the doctor who registered the patient.
3. The allocation history for the entire study is managed by a person with no involvement in patient enrollment, the interventions, or statistical analysis.
4. Withdrawal of consent, discontinuation, dropping out, and other such occurrences are immediately reported to the Study Coordinator.

## Allocation Method and Allocation Adjustment Factors

Subjects are randomly allocated to treatment groups by the Study Coordinator at the time they are registered as study participants. To prevent any major bias in (1) age, (2) sex, (3) subjective symptoms, or other factors in the process randomization, the minimization method is used with these factors as adjustment factors.

1. **Study Design**

## Content of Crossover Study


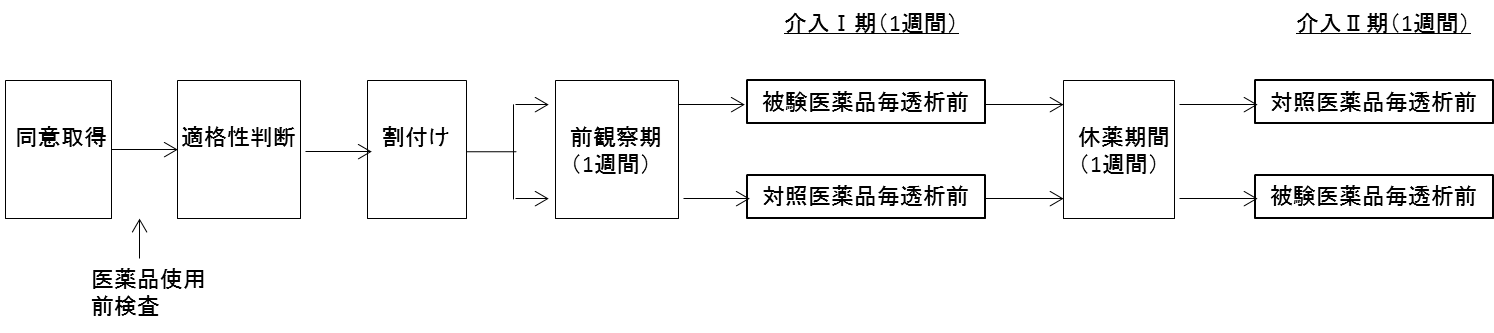


| 介入I期（1週間） | Period I (1 week) |
| --- | --- |
| 介入II期（1週間） | Period II (1 week) |
| 同意取得 | Consent obtained |
| 医薬品使用前検査 | Tests prior to investigational drug use |
| 適格性判断 | Eligibility determination |
| 割付け | Allocation |
| 前観察期（1週間） | Period 0 (measurement of baseline 1) (1 week) |
| 被験医薬品毎透析前 | Test drug use (before each dialysis procedure) |
| 対象医薬品毎透析前 | Control drug use ( before each dialysis procedure) |
| 休薬期間（1週間） | Wash-out period (1 week) |

(1) Written consent is obtained from the subject.

（2） Tests prior to investigational drug use are conducted, and eligibility for enrollment in the study (selection criteria/exclusion criteria) is confirmed.

(3) Subjects determined to be eligible as a result of the tests before investigational drug use are randomly allocated into two groups (Group A and Group B). Those in Group A use the test drug (EMLA) followed by the control drug (Youpatch), and those in Group B use the control drug (Youpatch) followed by the test drug (EMLA).

(4) In Period0 (1 week), pain is evaluated during arteriovenous fistula puncture using a visual analog scale (VAS) on all three occasions dialysis is performed from the start of the week to the weekend (Monday, Wednesday, and Friday or Tuesday, Thursday, and Saturday) without the use of the investigational drugs. The pain score (VAS) during arteriovenous fistula puncture during the final dialysis of that week is defined as the Baseline 1 pain score during arteriovenous fistula puncture (without drug use) [VAS (Group A pre-intervention Period I value) and VAS (Group B pre-intervention Period I value]. Health-related quality of life (QOL) is assessed using the SF36 (Acute Version: 1-week recall period) after the final dialysis of that week (Friday or Saturday).

（5）In Period I (1 week), the allocated investigational drug [Group A: test drug (EMLA); Group B: control drug (Youpatch)] is used on all three occasions dialysis is performed from the start of the week to the weekend (Monday, Wednesday, and Friday or Tuesday, Thursday, and Saturday). The pain score is evaluated by VAS after arteriovenous puncture on each occasion dialysis is performed. The mean pain score (VAS) during these three arteriovenous fistula punctures is defined as the pain score during arteriovenous fistula puncture when the drug concerned is used [Group A: VAS (Group A EMLA); Group B: VAS (Group B Youpatch)]. In addition, ΔVAS (Group A EMLA) [= VAS (Group A EMLA) – VAS (Group A pre-Intervention Period I score)] is defined as the total effect of EMLA Cream in Group A, and ΔVAS (Group B Youpatch) [= VAS (Group B Youpatch) – VAS (Group B pre-Intervention Period I score)] is defined as the total effect of Youpatch Tape in Group B. Health-related quality of life (QOL) is assessed using the SF36 (Acute Version: 1-week recall period) after the final dialysis of that week (Friday or Saturday).

(6) In the wash-out period (1 week), pain during arteriovenous fistula puncture is evaluated by VAS on all three occasions dialysis is performed from the start of the week to the weekend (Monday, Wednesday, and Friday or Tuesday, Thursday, and Saturday) without the use of the investigational drugs. The pain score (VAS) during arteriovenous fistula puncture during the final dialysis of that week is defined as the Baseline 2 pain score during arteriovenous fistula puncture (without drug use) [VAS (Group A pre-Intervention Period II score) and VAS (Group B pre-Intervention Period II score]. Health-related quality of life (QOL) is assessed using the SF36 (Acute Version: 1-week recall period) after the final dialysis of that week (Friday or Saturday).

(7) In Period II (1 week), use the allocated investigational drug [Group A: control drug (Youpatch); Group B: test drug (EMLA)] on all three occasions dialysis is performed from the start of the week to the weekend (Monday, Wednesday, and Friday or Tuesday, Thursday, and Saturday). The pain score is evaluated by VAS after arteriovenous puncture on each occasion dialysis is performed. The mean pain score (VAS) during these three arteriovenous fistula punctures is defined as the pain score during arteriovenous fistula puncture when the drug concerned is used [Group A: VAS (Group A Youpatch); Group B: VAS (Group B EMLA)]. In addition, ΔVAS (Group A Youpatch) [= VAS (Group A Youpatch) – VAS (Group A pre-Period II score)] is defined as the total effect of Youpatch Tape in Group A, and ΔVAS (Group B EMLA) [= VAS (Group B EMLA) – VAS (Group B pre- Period II score)] is defined as the total effect of EMLA in Group B. Health-related quality of life (QOL) is assessed using the SF36 (Acute Version: 1-week recall period) after the final dialysis of that week (Friday or Saturday).

## Criteria for Dose and Schedule Changes

The doses of the investigational drugs will not be changed in this study.

## Discontinuation of Treatment

The administration of an investigational drug is discontinued in the event that the Principal Investigator or another investigator determines that the study cannot be continued for one of the reasons below. The date of discontinuation, the reason, and the course are recorded in the medical record and the Case Report Form (CRF), the necessary tests at the point of discontinuation/dropping out are conducted, and efficacy and safety evaluations are performed.

If the investigational drug is discontinued because of an adverse event, if possible, the patient is followed up until recovery.

1. Notice from the study subject of withdrawal from the study or withdrawal of consent
2. Found not to meet the eligibility criteria after registration
3. Unable to continue study due to adverse event
4. Found to be pregnant
5. Subject unable to attend hospital due to moving house or similar reason
6. Study discontinuation determined to be appropriate by a doctor for any reason other than the discontinuation of the study as a whole

## Concomitant Treatment and Supportive Therapy

The subjects of this study are chronic maintenance hemodialysis patients, and the study is conducted on the basis of continuing treatment for chronic kidney failure and its complications.

## Post-Treatment

There are no stipulations on post-treatment.

1. **Parameters to be Monitored, Tested, and Reported, and Schedule**

## Parameters to be Monitored and Tested and Treatment Information to be Reported

**Consent Obtained – Determination of Eligibility for Study Enrollment**

Subject data: subject ID, age, sex, dialysis history, height, weight, complications, previous medical history, history of current condition, allergies, PS (ECOG), blood pressure, temperature, heart rate, etc.

**Pre-observation Period (1 week)**

The pain score during arteriovenous fistula puncture is measured by VAS (on every occasion dialysis is performed)

Health-related QOL is measured using the SF36 (Acute Version) (after final dialysis)

**Intervention Period I (1 week)**

The pain score during arteriovenous fistula puncture is measured by VAS (on every occasion dialysis is performed)

Health-related QOL is measured using the SF36 (Acute Version) (after final dialysis)

A check is made for drug-induced adverse events such as rash

**Drug Holiday Period (1 week)**

The pain score during arteriovenous fistula puncture is measured by VAS (on every occasion dialysis is performed)

Health-related QOL is measured using the SF36 (Acute Version) (after final dialysis)

A check is made for drug-induced adverse events such as rash

**Intervention Period II (1 week)**

The pain score during arteriovenous fistula puncture is measured by VAS (on every occasion dialysis is performed)

Health-related QOL is measured using the SF36 (acute version) (after final dialysis)

A check is made for drug-induced adverse events such as rash

## 8.2. Schedule for Monitoring, Testing, and Reporting

| Period  Parameter | Consent obtained – determination of eligibility for study enrollment | Pre-observation period (1 week) | Period I (1 week) | Wash out (1 week) | Period II (1 week) |
| --- | --- | --- | --- | --- | --- |
| Explanation of study/obtaining consent | ○ |  |  |  |  |
| Investigation of patient characteristics | ○ |  |  |  |  |
| Examination by doctor | ○ |  |  |  |  |
| Height, weight | ○ |  |  |  |  |
| Temperature, blood pressure, heart rate | ○ | ○ | ○ | ○ | ○ |
| Pain score during arteriovenous fistula puncture measured by VAS (on every occasion dialysis is performed) |  | ○ | ○ | ○ | ○ |
| Health-related QOL measured using the SF36 (acute version) (after final dialysis in that period) |  | ○ | ○ | ○ | ○ |
| Check made for adverse events caused by the interventional drug |  |  | ○ | ○ | ○ |
|  | | | | | |

8.3. Specimen Storage and Use of Specimens by Other Institutions, Etc.

Materials concerning this study stored by the study sites and the Principal Investigator (including records of subject observations, test data, Ethics Committee screening records, and subject consent forms) shall be retained until a date 5 years from the date of completion of the study as a whole or 3 years from the date of final publication of the results of the study, whichever is the later. After the end of the retention period, they are to be disposed of appropriately (shredded or burned).

Specimens such as blood and urine samples will not be collected in this study.

1. **Target Enrollment and Study Period**
   1. Target Enrollment

The target number of applications in this hospital is 20 patients.

The total number of subjects to be enrolled in the study as a whole is 68 patients.

(The study as a whole will include Kanazawa Medical University Hospital, Keiju Medical Center, Anamizu General Hospital, and others)

## Study Period

Registration period: From approval to December 31, 2018

Date of study termination: December 31, 2019

1. **Evaluation and Reporting of Adverse Events**

## Definition of Adverse Events

An adverse event is any undesirable or unintended injury or its manifestation (including abnormal clinical laboratory test results) that occurs in a study subject, irrespective of whether or not there is a causal relationship with an investigational drug. A side effect (adverse drug reaction, ADR) is an adverse event for which a causal relationship with an investigational drug cannot be excluded (assessed as having a “possible” causal relationship with the investigational drug).

Unexpected ADRs are those ADRs that are not listed on the package insert, or which are listed but for which the nature and severity are not consistent with the description given in the package.

## Evaluation and Reporting of Adverse Events

Any adverse events occurring within 30 days of the completion of the protocol treatment are to be reported to the Principal Investigator and the Study Coordinator.

The criteria for the severity grading of side effects are 1) Mild: Administration can be continued without treatment; 2) Moderate: Administration can be continued with some sort of treatment; and 3) Severe: Administration should be discontinued or temporarily suspended. Use MedDRA/J (Medical Dictionary for Regulatory Activities/J) for grading adverse events.

The Principal Investigator shall report the state of progress of the study and the occurrence of any adverse events, problems, or other difficulties to the Directors of the study sites once a year.

## Expected Adverse Events

Side Effects of EMLA Cream

Major side effects

(1) Shock, anaphylaxis (adverse events observed overseas so frequency unknown)

(2) Impaired consciousness, tremors, convulsions (adverse events observed overseas so frequency unknown)

(3) Methemoglobinemia (adverse event observed overseas so frequency unknown)

Other side effects

|  | ≥10% | 0.1%–10% | Frequency unknown (self-reported overseas) |
| --- | --- | --- | --- |
| Neurological |  | Paresthesia | Dizziness, hypoesthesia, headache |
| Gastroenterological |  |  | Nausea, vomiting |
| Skin | Erythema | Flushing, pallor, induration, pruritis | Vesicles, rash, hives, contact dermatitis, eczema, burning sensation in the skin, skin hyperpigmentation |
| Other |  | Elevated ALT (GPT) | Hematoma, pain, discoloration, swelling, malaise |

Side Effects of Youpatch Tape

Major side effects

Shock, anaphylaxis (frequency unknown)

Other side effects

|  | Frequency unknown |
| --- | --- |
| Hypersensitivity | Reddening, pruritis, contact dermatitis, irritation, hives, sensation of heat |
| Skin | Pigment deposition, skin exfoliation |

## Urgent Reporting and Treatment of Adverse

Follow up adverse events occurring within 30 days of completion of the crossover study until they improve.

- - 1. Urgent Reporting

Should a serious adverse event occur

1. Should a serious adverse event occur, the Principal Investigator or other investigator shall provide appropriate treatment. An investigator shall immediately report this to the Principal Investigator, irrespective of any causal relationship with an investigational drug.
2. The Principal Investigator shall immediately report the serious adverse event concerned to the Director of the study site, and shall notify the supplier of the investigational drug.

Contact for emergency reporting:

Principal Investigator: Keiji FUJIMOTO

Department of Nephrology, Kanazawa Medical University

Address: 1-1 Daigaku, Uchinada, Kahoku, Ichikawa 920-0293

Tel: 076-286-2211 (extension 8448)

Fax: 076-286-2786

1. **Cost to Study Subjects and Anticipated Risks and Benefits**

## Cost to Study Subjects and Anticipated Risks

Study subjects may develop side effects of EMLA Cream and Youpatch Tape. If they participate in the study while unaware of an allergy, they may develop allergic reactions such as cutaneous symptoms, mucosal symptoms, respiratory symptoms, gastrointestinal symptoms, neurological symptoms, circulatory symptoms, and anaphylactic shock.

## Anticipated Benefits to Study Subjects

Relief of pain during arteriovenous fistula puncture will improve QOL.

## Overall Assessment and Measures to Minimize Cost and Risk

Study subjects will be carefully selected to exclude those with allergies.

1. **Matters to be Reported to the Directors of Study Sites and Reporting Method**

During this study, the following matters are to be reported to the Directors of the study sites. The reporting method is stipulated by each institution.

## Reporting by Investigators and Subinvestigators

Investigators and subinvestigators shall report the following occurrences to the Directors of the study sites.

- The occurrence of a major concern from the standpoint of respecting the human rights of study subjects and others or concerning the implementation of the study, such as the leakage of data related to the study
- Circumstances or information that threaten the integrity of the conduct of the study or the reliability of the study results, or information that may potentially threaten these.

## Reporting by the Principal Investigator

The Principal Investigator shall report the following matters to the Directors of the study sites, and if necessary, shall consider suspending or terminating the study or amending the Study Protocol.

- Circumstances or information that threaten the ethical validity or the scientific rationality of the study, or information that potentially threaten these, which may affect the continuation of the study
- Circumstances or information that threaten the integrity of the conduct of the study or the reliability of the study results, or information that may potentially threaten these.
- The state of progress of the study
- Occurrences of adverse events associated with the study
- (For invasive studies) The occurrence of a serious adverse event
- Completion of the study (including termination)
- Prepare and submit a Study Completion Report with an overview of the study results
- [For invasive interventional studies (excluding minor invasions)] Final publication of the results
- Management status of human-derived samples and data

1. **Definitions of Endpoints**

## Primary Endpoint

Improvement in pain during arteriovenous fistula puncture before and after drug use (improvement in VAS score)

## Secondary Endpoint

Change in an overall indicator of Health-related QOL [SF36 (Acute Version) before and after drug use

1. **Statistical Discussion**
   1. Rationale for Determination of Target Enrollment

On the hypothesis that the use of EMLA Cream will increase VAS score by 10 mm compared with Youpatch Tape and an intra-individual standard deviation in VAS improvement of 20 mm, and with the α for type 1 error (α error) as 5% on both sides and 80% power (1 – β), 31 individuals are required in each group (62 in both groups). Assuming a discontinuation/dropout rate of 5%–10% due to drug-induced side effects and other reasons, the scheduled enrollment was set at 34 individuals per group (68 in both groups).

- 1. Analysis Populations

1. The analysis of the primary and secondary endpoints using the Full Analysis Set constitutes the primary analysis. An analysis using the Per Protocol Set (PPS) will also be performed to confirm the reliability of the analysis results.
   1. Analysis of Endpoints and Methods

Endpoints for analysis

1. Primary endpoint: Comparison of the analgesic effects of EMLA Cream and Youpatch Tape during arteriovenous fistula puncture
2. Secondary endpoint: Change in Health-related QOL [SF36 subscale score (0–100 points)] before and after the use of EMLA Cream and Youpatch Tape

**Method of Analysis of Primary Endpoint**

The primary endpoint is analyzed by the standard statistical procedures^18)^ for a two-group, two-period crossover study.

Total effect of EMLA Cream in Group A (E_A1I_) = ΔVAS (Group A EMLA)

Total effect Youpatch Tape in Group A (E_A2C_) = ΔVAS (Group A Youpatch)

Total effect of EMLA Cream in Group B (E_B2I_) = ΔVAS (Group B EMLA)

Total effect Youpatch Tape in Group B (E_B1C_) = ΔVAS (Group B Youpatch)

Crossover design analysis

|  | Period I | Period II | Difference in effects | Sum of effects |
| --- | --- | --- | --- | --- |
| Group A | EMLA  E_A1I_ = T_I_ + P_I_ + TP_A_ | Youpatch  E_A2C_ = T_C_ + P_2_ + TP_A_ | D_A(1 – 2)_ = E_A1I_ – E_A2C_  = (T_1_ – T_C_） + (P_1_ – P_2_) | S_A(1 + 2)_ = E_A1I_ + E_A2C_ = (T_I_ + T_C_) + (P_1_ + P_2_) + 2TP_A_ |
| Group B | Youpatch  E_B1C_ = T_C_ + P_1_ + TP_B_ | EMLA  E_B2I_ = T_I_ + P_2_ + TP_B_ | D_B(1 – 2)_ = E_B1C_ – E_B2I_  = [ – (T_I_ – T_C_)] + (P_1_ – P_2_) | S_B(1 + 2)_ = E_B1C_ + E_B2I_ = (T_I_ + T_C_) + (P_1_ + P_2_) + 2TP_B_ |

E: Total effect: T: treatment effect; P: period effect; TP: treatment–period interaction effects (or allocation effects or carryover effects).

Subscripts: I: EMLA Cream; C: Youpatch Tape; 1: Intervention Period I; 2: Intervention Period II; A: Group A; B: Group B.

(1) Analysis of period effect

D_A(1 – 2)_ – ( – D_B(1 – 2)_) = D_A(1 – 2)_ – D_B(2 – 1)_ = 2(P_1_ – P_2_)

Test whether or not this is 0 by comparing D_A(1 – 2)_ and D_B(2 – 1)_ using an unpaired t-test, and if the difference is not significant the period effect may be ignored.

(2) Analysis of carryover effect

S_A(1 + 2)_ – S_B(1 + 2)_ = 2(TP_A_ – TP_B_)

Test whether or not this is 0 by comparing S_A(1 + 2)_ and S_B(1 + 2)_ using an unpaired t-test, and if the difference is not significant the carryover effect may be ignored.

(3) Analysis of treatment–period interaction effects (or allocation effects or carryover effects)

D_A(1 – 2)_ – D_B(1 – 2)_ = 2(T_I_ – T_C_)

Compare D_A(1 – 2)_ and D_B(1 – 2)_ using an unpaired t-test, and test the difference between the treatment effect of EMLA Cream (T_I_) and that of Youpatch Tape (T_C_) for significance.

**Method of Analysis of Secondary Endpoint**

Analysis of the change in health-related QOL [SF36 subscale score (0–100 points)] before and after treatment

The analysis will be carried out in the same way as for the primary endpoint.

1. **Monitoring**
   1. Conduct of Monitoring

Monitoring will be carried out to confirm that this study is being conducted safely and in accordance with the protocol and that data are being collected accurately. Monitoring will be carried out on the basis of the data recorded in Case Report Forms by the Kanazawa Medical University Clinical Trial Center in accordance with the institution's Monitoring Manual.

- 1. Specific Procedures for Monitoring

1) A monitor belonging to the Kanazawa Medical University Clinical Trial Center will be responsible for monitoring this study.

2) After the first patient has been registered, monitoring will be conducted to check eligibility, the method of obtaining consent, and the registration method. If this reveals problems, corrective action will be taken. Subsequently, unless any specific matters of concern arise, monitoring will be carried out after every 10th patient registration or every 12 months.

3) Monitoring will also be conducted as required should an unexpected serious adverse event occur or in cases with a major effect on the performance of the study.

4) The timing and nature of its conduct will be reviewed in accordance with the state of progress of monitoring.

5) The monitor will submit a Monitoring Report to the Principal Investigator immediately after monitoring has been conducted.

- 1. Matters to be Monitored

1. Confirmation of consent and eligibility
2. Status of implementation of the study
3. Protocol deviations
4. Serious adverse events
5. Adverse events and other problems concerning the progress and safety of the study
6. **Ethical Matters**
   1. Regulations to be Observed

All persons involved in this study must have thoroughly read and understood the WMA Helsinki Declaration and the Ethical Guidelines for Medical and Health Research Involving Human Subjects (MEXT/MHLW), to which all medical research on human subjects must conform, and comply with them in the conduct of the study.

- 1. Preparation and Revision of Patient Information Sheets and Consent Forms

A Consent Form is a document indicating a study subject's intention to participate in a clinical study.

In accordance with the Ethical Guidelines for Medical and Health Research Involving Human Subjects and Article 51 of the GCP Ordinance, a Consent Form shall be produced and completed by all the study subjects. It will be revised if necessary.

- 1. Informed Consent

When obtaining informed consent, in principle the following matters must be explained to the study subjects and others concerned. However, this is with the exception of matters authorized by the Director of the study site, in light of the opinion of the Ethics Committee.

(1) The study title and the fact that the conduct of this study has been authorized by the Director of the study site

(2) The names of the study site and the Principal Investigator (including the names of other sites jointly conducting the study and their Principal Investigators if the study is being conducted jointly with other study sites)

(3) The purpose and significance of the study

(4) The study methods (including the purpose of use of any samples or data obtained from the study subjects) and the study period

(5) The reason for selection as a study subject

(6) The costs to study subjects and anticipated risks and benefits

(7) That subjects may withdraw their consent at any time after starting to participate in or continuing the study (if it may be difficult to take measures in line with the nature of the withdrawal by the study subject or other persons concerned, this fact and the reason)

(8) That the subject or other persons concerned will not be placed at a disadvantage if they do not consent to participate in or continue the study or if they withdraw their consent

(9) How information on the study will be disclosed

(10) That the study subject or other persons concerned may request to be given or view the Study Protocol or documentation on the study methods, insofar as this does not interfere with protecting the personal information of other study subjects and other persons concerned or with assuring the originality of the study concerned, and how to obtain or view these documents

(11) Handling of personal and other information (including the anonymization method if used)

(12) Methods of storage and disposal of specimens and data

(13) Conflicts of interest for the study site concerning this study, such as funding sources, and individual conflicts of interest for investigators and subinvestigators concerning this study, such as personal income

(14) How requests for advice from study subjects, other persons concerned, and their friends and relatives will be dealt with

(15) Details of any economic cost to or honorarium provided for study subjects and other persons concerned

(16) For studies involving medical actions beyond the scope of regular medical care, alternative treatment methods and related matters

(17) For studies involving medical actions beyond the scope of regular medical care, the provision of medical care to study subjects after their participation in the study

(18) For studies involving invasive procedures, details of any compensation for health damage resulting from the study

(19) For studies involving invasive interventions (other than those of minor invasiveness), the fact that if necessary, persons engaged in monitoring and auditing and members of the Ethics Committee will have access to specimens and data obtained from subjects of the study, on the basis that subjects' confidentiality will be preserved

1. **Handling of Personal Information**

A subject ID will be used to identify patients in patient registration and Case Report Forms, and in the conduct of the study every effort will be made to protect patients' privacy with respect to direct access to original data and patient consent forms and other documents and in the publication of the study results. Study personnel shall comply with applicable legislation and regulations concerning the protection of study subjects' personal information. Persons engaged in the study shall also make every effort to protect study subject' personal information and privacy, and personal information obtained in the performance of the study may not be leaked without justifiable reason. This still applies after study personnel have left their jobs.

1. **Study Costs**
   1. Source of Funding and Financial Relationships

This study is carried out with research funding from the Department of Nephrology of Kanazawa Medical University. In the conduct and publication of this study, conflicts of interest shall be managed appropriately, maintaining neutrality and impartiality and keeping the study fair. When publishing the study results, the authors will comply with the directives of the conference or journal in which the results are published, self-disclosing the situation accurately.

The planning, conduct, and reporting of this study shall be carried out appropriately in accordance with the Kanazawa Medical University Conflict of Interest Management Regulations. It will also be confirmed that the conduct of the study does not infringe the rights or benefits of the subjects.

- 1. Costs Associated with the Study

The costs of the interventional drugs used in this study are within the scope of regular clinical practice, and will be borne by the patient (under health insurance). The cost of the right to use the SF36 will be covered by the Department of Nephrology research funds.

- 1. Compensation for Health Damage

Because all the drugs used in this study are commercially available and will be used in accordance with the package insert (indications and dosage/administration), should a serious side effect appear it will be treated as if it occurred during regular clinical practice. They are also eligible for relief benefit applications under the Drug Side Effect Damage Relief System.

1. **Protocol Deviations**

## Deviations from or Changes to the Study Protocol

The Principal Investigator and other investigators may not deviate from or change the Study Protocol without the advance agreement of the Study Chair and the approval of the Hospital Director based on advance screening by the Clinical Research Ethics Committee.

The Principal Investigator and other investigators may deviate from or change the Study Protocol before receiving the agreement of the Study Chair and the approval of the Clinical Research Ethics Committee if this is necessary to avoid an emergency. In this case, the Principal Investigator or other investigator shall immediately submit a description of the deviation or change and its reason, as well as proposed revisions to the Study Protocol and other documentation if required, to the Study Chair and the Clinical Research Ethics Committee, and shall obtain the approval of the Study Coordinator, the Clinical Research Ethics Committee, and the Hospital Director.

In the event that a deviation from the Study Protocol has occurred, the Principal Investigator and other investigators must record all such deviations and their reasons.

## Revisions to the Study Protocol

The Principal Investigator shall submit the Study Protocol to the Directors of the study sites and obtain approval from the Clinical Research Ethics Committee and the Directors of the study sites before the start of the study.

When revising the Study Protocol, the Principal Investigator shall decide on revisions after consulting if necessary with the Statistical Analysis Manager concerning the validity of the changes and their effect on the evaluation of the study. When making revisions, they must submit the revised Protocol to the Directors of the study sites and obtain approval from the Clinical Research Ethics Committee and the Directors of the study sites. In the event of major changes, a temporary suspension of patient registration must be considered from the viewpoint of protecting the study subjects.

After obtaining approval for the revisions, the Principal Investigator shall immediately inform the other investigators, the Data Center, and other persons involved in the study of the content of the revisions.

1. **Termination or Early Completion of the Study**

## Completion of the Study

When the study is completed at each site, the Principal Investigator shall immediately submit a Study Completion Report to the Director of the study site. If the study is conducted at multiple sites, they shall also submit the Study Completion Report to the Study Chair and the Study Coordinator.

## Early Termination of the Study

The Principal Investigator shall consider whether or not to continue the study should any of the following apply.

1. Important information has been received concerning the quality, safety, or efficacy of the investigational drugs
2. Due to difficulty in recruiting study subjects, it is considered that it will be completely impossible to achieve the target enrollment
3. The Clinical Research Ethics Committee instructs that changes be made to the Protocol which are considered to be unacceptable
4. **Handling of Materials Associated with Study Materials**

The Principal Investigator shall store documents concerning the conduct of the study and related matters (copies of application documents, notifications from Hospital Directors, copies of application forms and reports, the subject ID list, consent forms, copies of Case Report Forms and related documentation, and other documents and records required to guarantee the reliability of the data), and shall destroy them 5 years after study publication.

1. **Ownership of Study Results and Publication of Effects**

This study will be registered with the University Hospital Medical Information Network (UMIN) before the start of the trial.

The results of this study will belong to the Research Group. The Principal Investigator (lead investigator) will be the lead author when the results are reported at a conference or as a paper.

1. **Study Organization**

## Study Sites

Multicenter joint study: Lead study site

Department of Nephrology, Kanazawa Medical University

1-1 Daigaku, Uchinada, Kahoku, Ichikawa 920-0024

Tel: 076-286-2211 (switchboard) (extension 8448)

Tasks: Data collection, anonymization, measurements, data analysis

Joint study sites (anticipated)

Department of Nephrology, Keiju Medical Center

Department of Nephrology, Anamizu General Hospital

## Principal Investigator

Keiji FUJIMOTO, Department of Nephrology, Kanazawa Medical University

## Study Coordinator

1-1 Daigaku, Uchinada, Kahoku, Ichikawa 920-0293

Contact: Keiji FUJIMOTO

Tel: 076-237-2211 (extension 8448)

Fax: 076-286-2786

E-mail: k-2210@kanazawa-med.ac.jp

## Statistical Analysis Manager

Yasuo IIDA, Associate Professor, Department of Natural Sciences (Mathematics), Division of General Education, Kanazawa Medical University

## Data Center and Data Manager

Data Center

Department of Nephrology, Kanazawa Medical University

Address: 1-1 Daigaku, Uchinada, Kahoku, Ichikawa 920-0293

Tel: 076-286-2211 (extension 8448)

Fax: 076-286-2786 Contact person

Data Manager

Keiji FUJIMOTO, Department of Nephrology, Kanazawa Medical University

## Monitoring

Clinical Trial Center, Kanazawa Medical University

Address: 1-1 Daigaku, Uchinada, Kahoku, Ichikawa 920-0293

Tel: 076-286-2211

1. **Support Desk**

Keiji FUJIMOTO, Department of Nephrology, Kanazawa Medical University

Address: 1-1 Daigaku, Uchinada, Kahoku, Ichikawa 920-0293

Tel: 076-286-2211 (extension 8448)

Fax: 076-286-2786

1. **References**
2. Nariyama S, Kamioka M, Yamane T, Kuroki K, Kawano Y, Miyauji R, Kosaka F, Shiroki A, Kitajima S, Murakami N, Ogata S, Hayashi H, Anraku H, Hamamatsu H, Shibuya K. Two cases suggesting that lidocaine/propitocaine cream may be effective for pain relief during vascular access puncture in maintenance hemodialysis patients. Jpn J Clin Dialysis (0910–5808) 32(8): 1096–1102 (2016.07) [In Japanese]
3. Sherwood KA. The use of topical anesthesia in removal of port-wine stains in children. J Pediatr. May; 122(5 Pt 2): S36–40, 1993.
4. McCafferty DF, Woolfson AD, Handley J, Allen G. Effect of percutaneous local anaesthetics on pain reduction during pulse dye laser treatment of portwine stains. Br J Anaesth. Mar; 78(3): 286–9,1997
5. Hanaoka K, Watanabe S. A comparative clinical trial of lidocaine and propitocaine eutectic cream (SKA-01) in patients undergoing skin laser treatment: A multicenter placebo-controlled randomized double-blind parallel-group comparative trial. J Clin Therap Med 28(4): 279–291 (2012.04) [In Japanese]
6. Attal N, Brasseur L, Chauvin M, Bouhassira D. Effects of single and repeated applications of a eutectic mixture of local anaesthetics (EMLA) cream on spontaneous and evoked pain in post-herpetic neuralgia. Pain. 1999; 81(1–2):

203–9.

1. Hallén B, Uppfeldt A. Does lidocaine–prilocaine cream permit painfree insertion of IV catheters in children? Anesthesiology. 1982; 57(4): 340–2.
2. Maunuksela EL, Korpela R. Double–blind evaluation of a lignocaine–prilocaine cream (EMLA) in children. Effect on the pain associated with venous cannulation. Br J Anaesth. 1986; 58(11): 1242–5.
3. Manner T, Kanto J, Iisalo E, Lindberg R, Viinamäki O, Scheinin M. Reduction of pain at venous cannulation in children with a eutectic mixture of lidocaine and prilocaine (EMLA cream): comparison with placebo cream and no local premedication. Acta Anaesthesiol Scand. 1987; 31(8): 735–9.
4. Hallén B, Olsson GL, Uppfeldt A. Pain-free venepuncture. Effect of timing of application of local anaesthetic cream. Anaesthesia. 1984;39(10):969–72.
5. Hui-Chen F, Hsiu-Lin C, Shun-Line C, Tai-Ling T, Li-Jung W, Hsing-I T, San-Nan Y. The effect of EMLA cream on minimizing pain during venipuncture in premature infants. J Trop Pediatr. 2013; 59(1): 72–3.
6. Akdas O, Basaranoglu G, Ozdemir H, Comlekci M, Erkalp K, Saidoglu L. The effects of Valsalva maneuver on venipuncture pain in children: comparison to EMLA(®) (lidocaine–prilocaine cream). Ir J Med Sci. 2014;183(4):517–20.
7. Cooper CM, Gerrish SP, Hardwick M, Kay R. EMLA cream reduces the pain of venepuncture in children. Eur J Anaesthesiol. 1987; 4(6): 441–8.
8. Hopkins CS, Buckley CJ, Bush GH. Pain-free injection in infants. Use of a lignocaine–prilocaine cream to prevent pain at intravenous induction of general anaesthesia in 1–5-year-old children. Anaesthesia. 1988;43(3):198–201.
9. Hanaoka K, Okubo A. Efficacy and safety of lidocaine and propitocaine eutectic cream (SKA-01) in patients scheduled to undergo venepuncture: A multicenter placebo-controlled randomized double-blind parallel-group comparative trial. J Clin Therap Med 31(7): 683–697 (2015.07) [In Japanese]
10. Brodin A, Nyqvist-Mayer A, Wadsten T, Forslund B, Broberg F. Phase diagram and aqueous solubility of the lidocaine–prilocaine binary system. J Pharm Sci. Apr; 73(4): 481–4, 1984.
11. Nyqvist-Mayer AA, Brodin AF, Frank SG. Phase distribution studies on an oil-water emulsion based on a eutectic mixture of lidocaine and prilocaine as the dispersed phase. J Pharm Sci. Nov; 74(11): 1192–5, 1985
12. Çelik G, Özbek O, Yılmaz M, Duman I, Özbek S, Apiliogullari S. Vapocoolant spray vs lidocaine/prilocaine cream for reducing the pain of venipuncture in

hemodialysis patients: a randomized, placebo-controlled, crossover study. Int J Med Sci. ;8(7):623–7, 2011

1. Hills M, Armitage P. The two-period cross-over clinical trial. Br J Clin Pharmacol; 8(1): 7–20, 1979.
